# Exploring the Mechanism of Action of Chicoric Acid Against Influenza Virus Infection Based on Network Pharmacology, Molecular Docking, and Molecular Dynamics Simulation

**DOI:** 10.3390/ijms262210884

**Published:** 2025-11-10

**Authors:** Weijun Guo, Fuhao Ye, Zengyao Hou, Quanhai Pang

**Affiliations:** College of Veterinary Medicine, Shanxi Agricultural University, Jinzhong 030801, China; guoweijun@sxau.edu.cn (W.G.); yefuhao@sxau.edu.cn (F.Y.);

**Keywords:** Chicoric acid, network pharmacology, antiviral activity, target

## Abstract

This study theoretically explores the mechanism of action of Chicoric acid against influenza virus based on network pharmacology, molecular docking, and molecular dynamics simulation techniques, aiming to provide insights for the development of new veterinary drugs for influenza. Potential targets for influenza virus action were identified using the PharmMapper (i.e. Version 2017) server and disease databases including GeneCards and OMIM. The STRING online analysis platform and Cytoscape 3.9.1 software were employed to construct a protein–protein interaction (PPI) network of the target proteins, followed by topological analysis to screen for key targets. Gene Ontology (GO) enrichment analysis and Kyoto Encyclopedia of Genes and Genomes (KEGG) pathway enrichment analysis were performed on the intersecting targets using the DAVID database. A “drug–target–pathway” network diagram was constructed using Cytoscape 3.9.1 software. Molecular docking was carried out with AutoDock 1.5.6 and PyMOL 2.5 software to identify dominant binding targets, followed by molecular dynamics simulation analysis. The results of network analysis showed that there were 31 potential targets of Chicoric acid; the protein interaction network suggested that UBC, UBA52, RPS27A, HCK, and CDKN1B may be the core targets of Chicoric acid; 55 cell biological processes were obtained by GO enrichment analysis, and 15 related signaling pathways were obtained by KEGG pathway enrichment analysis; molecular docking showed that UBC and UBA52 had a good affinity to Chicoric acid and may be the dominant target of Chicoric acid exerting its effect. Chicoric acid may play a role in antiviral activity by acting on the dominant protein of UBC and UBA52, thus achieving an anti-influenza virus effect.

## 1. Introduction

As a representative pathogen of the Orthomyxoviridae family, the influenza virus exhibits broad host tropism and is capable of causing seasonal epidemics as well as periodic pandemics in both humans and animals [1]. Owing to its considerable zoonotic potential, this virus remains a persistent and serious threat to global public health [2]. Influenza A virus, in particular, presents substantial challenges for disease control, which are largely attributable to its high mutation rate and propensity to develop drug resistance. The continual emergence of resistant viral strains, coupled with the limited repertoire of antiviral drug targets, underscores the urgent need to develop novel and efficient antiviral agents with multi-target mechanisms of action. Given their multi-target activity and favorable toxicity profiles, natural products have thus emerged as a valuable source for identifying antiviral lead compounds in drug discovery.

Chicoric acid, a derivative of caffeic acid, is widely present in various medicinal plants such as Echinacea, chicory, dandelion, Clematis, and coneflower [3,4,5,6,7]. It exhibits a range of pharmacological properties, including antioxidant, free radical-scavenging [8,9], anti-inflammatory, antiviral, antibacterial, and immunomodulatory activities [10,11]. In recent years, its antiviral potential has garnered increasing attention due to its intrinsic capacity to target multiple key viral proteases and enzymes essential for viral replication—such as integrase and polymerase. For instance, in human immunodeficiency virus (HIV) studies, Chicoric acid has been demonstrated to effectively inhibit HIV integrase activity via hydrogen bonding and hydrophobic interactions, thereby preventing the integration of viral DNA into the host genome [12,13]. In severe acute respiratory syndrome coronavirus 2 (SARS-CoV-2) research, both molecular docking and in vitro experiments have confirmed that Chicoric acid binds with high affinity to the active sites of the main protease (Mpro/3CLpro) and papain-like protease (PLpro), thereby disrupting viral polyprotein processing and replication [14,15,16]. Furthermore, studies on dengue virus and hepatitis C virus (HCV) have indicated that Chicoric acid also exerts inhibitory effects on viral proteases such as NS2B-NS3 and NS3/4A [17,18,19]. Influenza virus replication similarly relies on key proteins including RNA polymerase subunits (PA, PB1, PB2) and neuraminidase (NA), suggesting that Chicoric acid may interfere with the influenza virus life cycle through analogous mechanisms.

Given the multifaceted bioactivities and therapeutic potential of Chicoric acid, along with the persistent need for effective influenza treatments, it is of significant importance to explore its anti-influenza mechanisms and application prospects. However, the precise molecular mechanisms underlying its anti-influenza activity remain incompletely elucidated. Therefore, this study aims to employ network pharmacology to predict potential targets and signaling pathways involved in the anti-influenza action of Chicoric acid. Molecular docking will be utilized to investigate the binding modes between Chicoric acid and key influenza-related target proteins, and molecular dynamics simulations will be applied to evaluate the stability of these interactions. This integrated approach seeks to clarify the anti-influenza mechanism of Chicoric acid at the molecular level and provide a theoretical foundation for the development of novel anti-influenza therapeutics.

## 2. Results

### 2.1. Screening of Targets for Chicoric Acid Against Influenza

A total of 287 potential targets of Chicoric acid were collected from the PharmMapper database, while 3671 influenza virus targets were obtained by integrating data from the GeneCards and OMIM databases. An intersection analysis of potential targets of Chicoric acid and influenza virus was conducted, identifying 31 common candidate targets (Table 1).

### 2.2. Venn Diagram of Chicoric Acid and Influenza Intersection Targets

**Figure 1:**
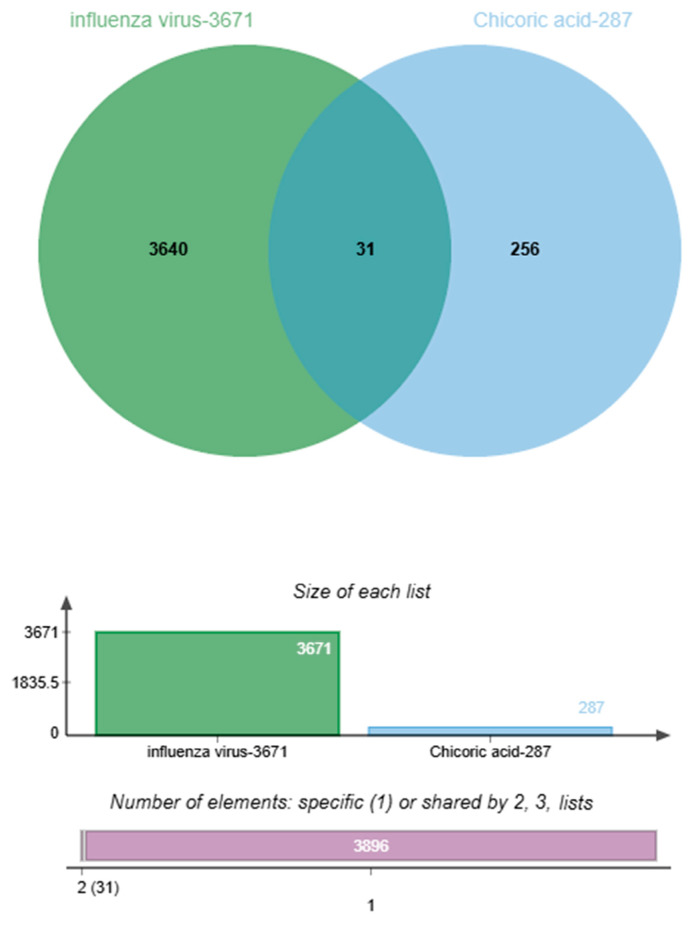
Intersection of Chicoric acid and anti-influenza virus targets.

This study used the proportional Venn diagram function of the bio-online mapping platform to comparatively analyze the targets of influenza disease and the targets of Chicoric acid, thereby identifying the anti-influenza targets of Chicoric acid (Figure 1).

### 2.3. Construction of Protein–Protein Interaction Networks

**Figure 2:**
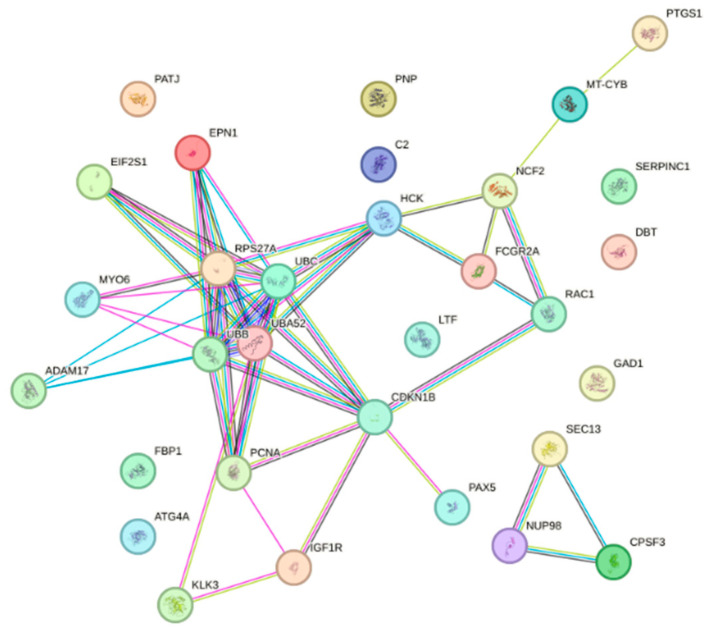
Potential target protein interaction network of Chicoric acid.

**Figure 3:**
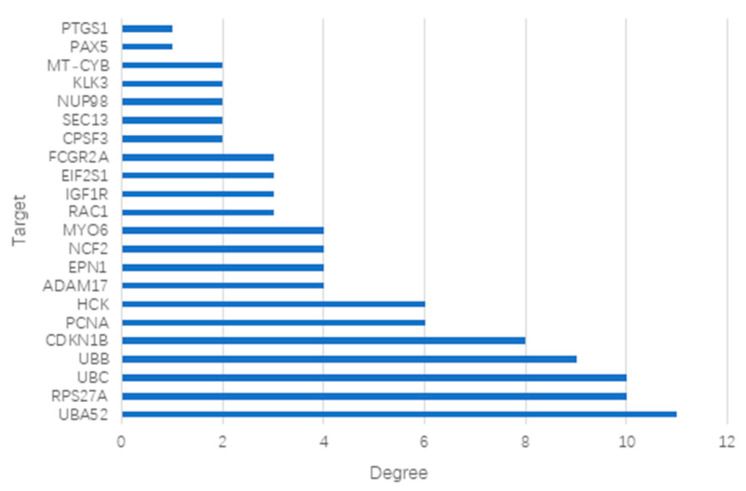
Order of anti-influenza virus potential target values of Chicoric acid.

**Figure 4:**
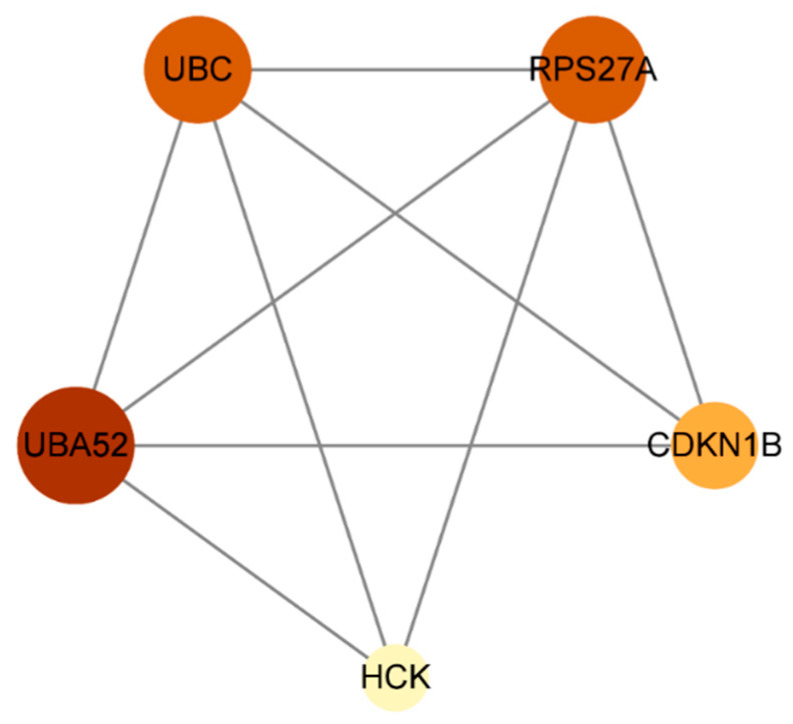
Hithub network of Chicoric acid anti-influenza virus target proteins.

The 31 screened potential anti-influenza targets of Chicoric acid were input into the STRING database with the species restricted to Homo sapiens, resulting in a protein–protein interaction (PPI) network (Figure 2). The network consisted of 31 nodes and 50 edges, with an expected number of edges of 41. The average node degree was 3.23, and the average local clustering coefficient was 0.464. The PPI enrichment *p*-value was 0.0831. The results were saved as a TSV file. The file was imported into Cytoscape 3.9.1 software, and a bar chart was generated based on the degree values (Figure 3). As shown in the figure, the top five targets were UBA52, RPS27A, UBC, UBB, and CDKN1B. Using the CentiScaPe 2.2 app within the software, a hubs network was constructed with node degree (degree = 4.947), betweenness centrality (betweenness = 22.526), and closeness centrality (closeness = 0.026) as screening criteria to identify core targets. The results are shown in Figure 4. The analysis revealed that the core targets of Chicoric acid against influenza are UBA52, UBC, HCK, CDKN1B, and RPS27A.

### 2.4. GO/KEGG Analysis Results

**Figure 5:**
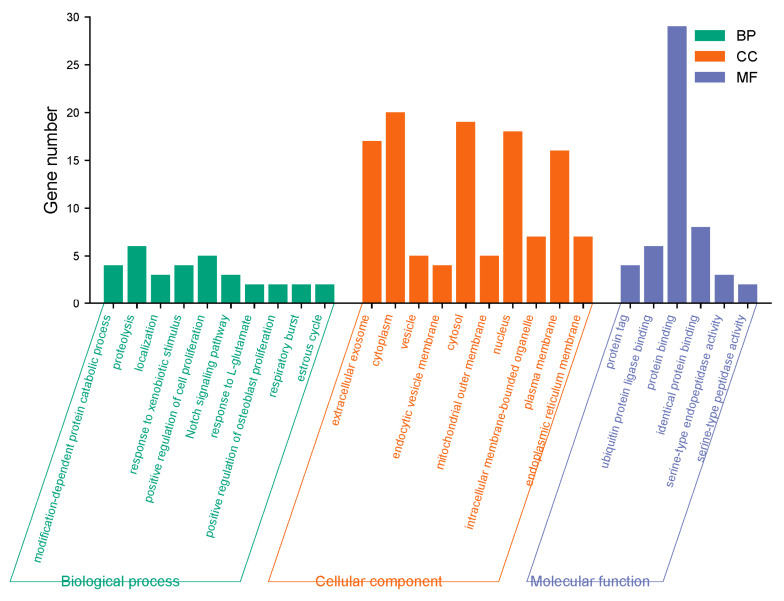
GO enrichment analysis of Chicoric acid anti-influenza virus target.

**Figure 6:**
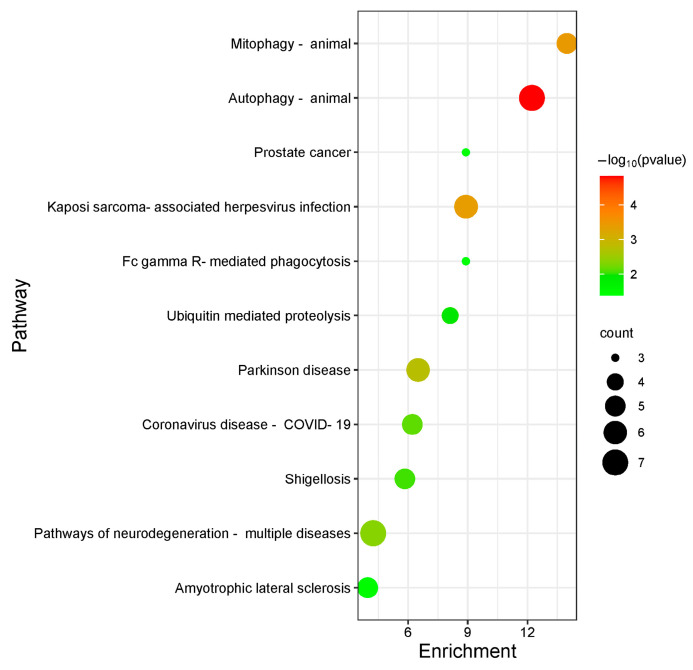
KEGG pathway analysis of Chicoric acid anti−influenza virus target.

The 31 screened targets of Chicoric acid against influenza virus were input into the DAVID database with the species limited to Homo sapiens for GO functional analysis. A total of 55 enrichment results were obtained, including 24 biological processes, 25 cellular components, and 6 molecular functions. Among them, the biological processes involved include the Notch signaling pathway, modification-dependent protein catabolic process, response to exogenous stimuli, positive regulation of cell proliferation, response to L-glutamate, and positive regulation of osteoblast proliferation. The molecular functions involved include protein binding, ubiquitin protein ligase binding, protein tagging, and serine-type endopeptidase activity. Cellular components involved include the cytoplasm, nucleus, extracellular exosome, plasma membrane, vesicle, endoplasmic reticulum membrane, and mitochondrial outer membrane. The top 10 processes ranked by *p*-value for biological processes, cellular components, and molecular functions in the GO functional analysis were plotted as a bar graph, with the results shown in Figure 5. KEGG pathway analysis yielded a total of 15 pathways. Bubble plots were generated for KEGG pathways with *p* < 0.05 using R packages from Bioconductor (Figure 6). The anti-influenza virus mechanisms of Chicoric acid primarily involve pathways such as autophagy, Kaposi’s sarcoma-associated herpesvirus infection, pathways of neurodegenerative diseases, shigellosis, and ubiquitin-mediated proteolysis.

### 2.5. Construction of the Chicoric Acid-Target-Pathway Network

**Figure 7:**
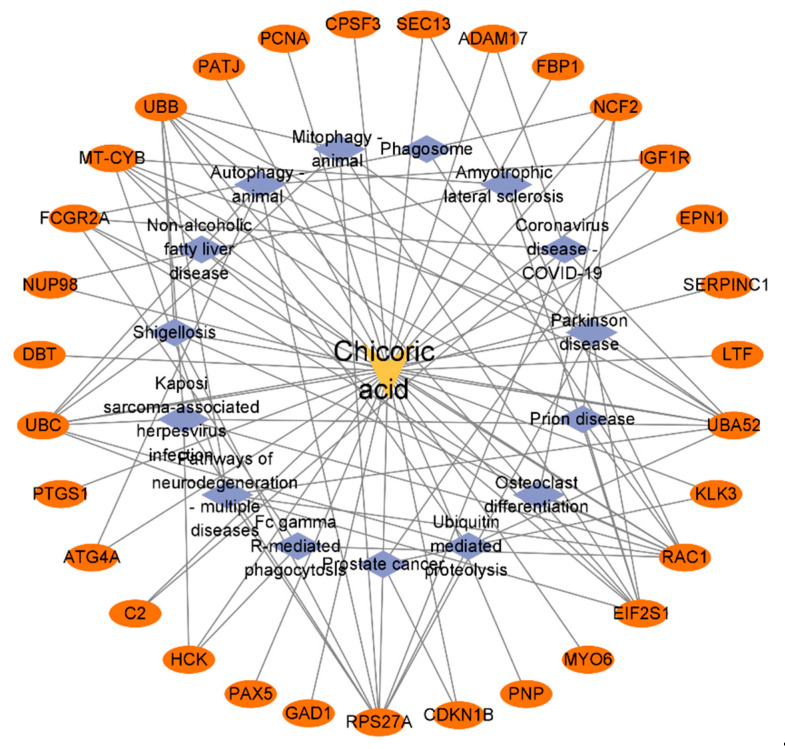
Network model of Chicoric acid–target–pathway.

The data obtained from DAVID pathway annotation was used to construct a Chicoric acid-target-pathway network model using Cytoscape 3.9.1 software, as shown in Figure 7. The Network Analyzer results indicated that the network consisted of 47 nodes and 100 edges, with a network density of 0.093, a network diameter of 4, and a network radius of 2. The network centralization was 0.607, network heterogeneity was 1.109, the characteristic path length was 2.331, and the average network degree was 4.255.

### 2.6. Molecular Docking Validation of Chicoric Acid

**Figure 8:**
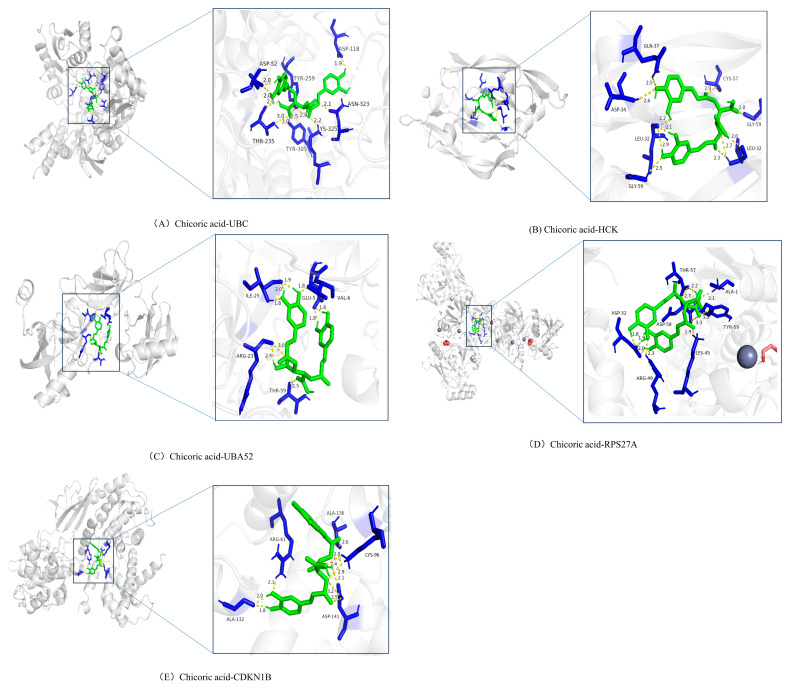
Molecular docking result of Chicoric acid with core target proteins. The green structures represent Chicoric acid, the blue structures indicate the binding sites of different receptor proteins with Chicoric acid, and the yellow dashed lines represent hydrogen bonds.

The five screened core targets (*UBA52*, *UBC*, *RPS27A*, *HCK*, and *CDKN1B*) were selected and subjected to molecular docking with Chicoric acid using AutoDock Tools 1.5.6 software (Table 2). It is generally accepted that a ligand and receptor can spontaneously bind when their binding energy is <0 kJ/mol, and when the binding energy is ≤−20 kJ/mol, they exhibit strong affinity. Although the binding energies of Chicoric acid with all five core targets were <0 kJ/mol, proteins UBC, HCK, and UBA52 demonstrated strong affinity with Chicoric acid, suggesting the potential formation of stable complexes. Thus, proteins UBC, HCK, and UBA52 may represent the dominant binding targets for Chicoric acid. Molecular docking diagrams revealed hydrogen bond interactions between Chicoric acid and all five target proteins. The theoretical binding modes are illustrated in Figure 8.

### 2.7. Molecular Dynamics Simulation Results

The top three complexes with the highest absolute binding energies with Chicoric acid (UBC, HCK, and UBA52) were selected for molecular dynamics simulation to analyze the stability of the binding between Chicoric acid and the protein targets, as well as the hydrogen bond interactions and binding interactions.

#### 2.7.1. Stability Analysis

##### RMSD Analysis

**Figure 9:**
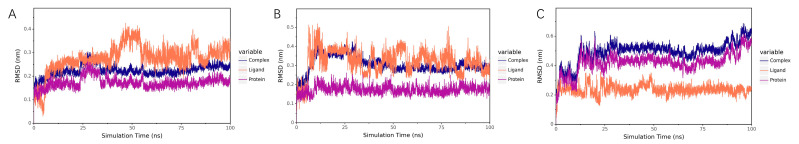
RMSD analysis of molecular dynamics simulation results of UBC, HCK, and UBA52 with Chicoric acid: (**A**) UBC, (**B**) HCK, and (**C**) UBA52.

RMSD (root mean square deviation) is a commonly used metric to quantify the differences between two structures. It is calculated by comparing the spatial coordinates of corresponding atoms in two molecules, reflecting the conformational similarity between them. It is used to track changes in molecular structure relative to the initial configuration during simulation and to observe whether the magnitude of change stabilizes over time. A lower RMSD value indicates closer structural similarity. As shown in Figure 9A–C, the RMSD values of proteins UBC, HCK, and UBA52 remained largely stable throughout the simulation. Similarly, the RMSD values of the complexes formed with Chicoric acid also remained stable during the simulation, indicating that the structures of the complexes remained generally stable.

##### Rg Analysis

**Figure 10:**
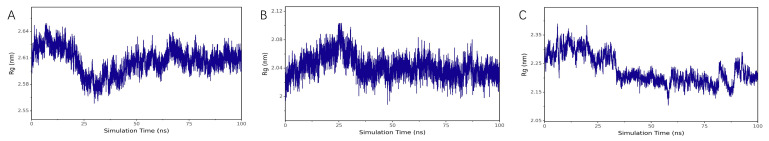
Rg analysis of molecular dynamics simulation results of UBC, HCK, and UBA52 with Chicoric acid: (**A**) UBC, (**B**) HCK, and (**C**) UBA52.

The radius of gyration (Rg) represents the root mean square distance of all atoms in a molecule relative to its center of mass, reflecting the spatial distribution range of atoms and serving as a key parameter to evaluate the structural compactness of protein–ligand complexes. A smaller Rg value indicates a more compact molecular structure, while a larger Rg suggests a looser, potentially disordered conformation. As shown in Figure 10A–C, the Rg values of the complexes formed between proteins UBC, HCK, and UBA52 and Chicoric acid gradually stabilized during the simulation, indicating that the structures of the complexes attained increasing stability.

##### RMSF Analysis

**Figure 11:**
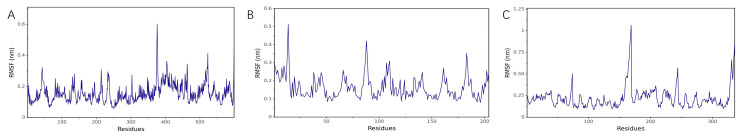
RMSF analysis of molecular dynamics simulation results of UBC, HCK, and UBA52 with Chicoric acid: (**A**) UBC, (**B**) HCK, and (**C**) UBA52.

Root mean square fluctuation (RMSF) quantifies the flexibility of amino acid residues in a protein by measuring the fluctuation of each atom or residue throughout the molecular dynamics trajectory. It reflects the displacement amplitude of each atom or residue relative to its average position, aiding in the identification of flexible and rigid regions within the molecule. As shown in Figure 11A–C, the simulation results are consistent with the crystal structure.

##### Centroid Evolution Analysis

**Figure 12:**
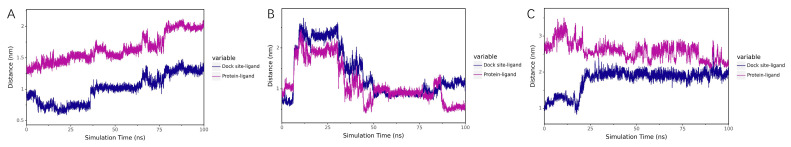
Distance evolution between ligand and protein binding pocket of UBC, HCK, and UBA52 with Chicoric acid: (**A**) UBC, (**B**) HCK, and (**C**) UBA52.

To analyze the state of the small molecule on the protein surface and identify its initial docking site, the distance between the centroid of residues at the initial docking site and the centroid of the small molecule was calculated. Additionally, the distance between the small molecule and the protein centroid was analyzed to evaluate the binding status. As shown in Figure 12A–C, the distances between Chicoric acid and the centroids of the three proteins (UBC, HCK, UBA52), as well as the distance between Chicoric acid and its initial binding site, gradually stabilized. This indicates that the small molecule stably binds to the initial binding site of the proteins, and the binding between the small molecule and the proteins progressively stabilizes.

##### Buried SASA Analysis

**Figure 13:**
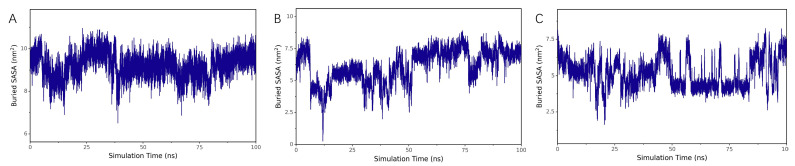
Buried SASA analysis of molecular dynamics simulation results of UBC, HCK, and UBA52 with Chicoric acid: (**A**) UBC, (**B**) HCK, and (**C**) UBA52.

Buried solvent-accessible surface area (Buried SASA) is a metric used to evaluate the regions within a molecule or molecular complex that are buried internally and inaccessible to solvent. A larger Buried SASA value indicates stronger intermolecular interactions and a larger contact area. As shown in Figure 13A–C, the Buried SASA values gradually stabilized, suggesting that the contact area between Chicoric acid and the three proteins (UBC, HCK, UBA52) became increasingly consistent, and the binding between them progressively stabilized.

##### Superposition of Binding Conformations

**Figure 14:**
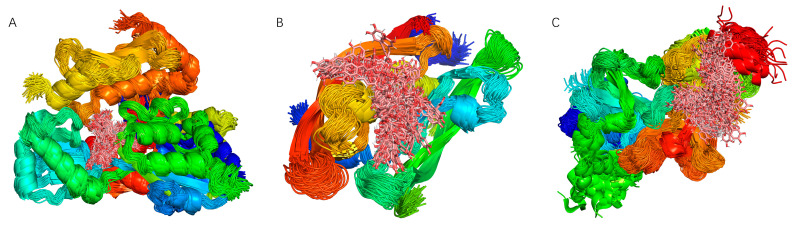
Superposition of binding conformation analysis of molecular dynamics simulation results of UBC, HCK, and UBA52 with Chicoric acid: (**A**) UBC, (**B**) HCK, and (**C**) UBA52.

The simulation trajectories were processed to superimpose the simulated conformations, with the results shown in Figure 14A–C. The high degree of conformational overlap for Chicoric acid indicates that the small molecule consistently remained bound to the initial binding site throughout the simulation.

#### 2.7.2. Analysis of Hydrogen Bond Interactions Between the Small Molecule and Proteins

##### Evolution of Hydrogen Bond Numbers

**Figure 15:**
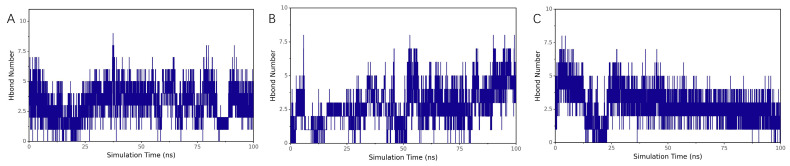
Hydrogen bond population dynamics of molecular dynamics simulation results of UBC, HCK, and UBA52 with Chicoric acid: (**A**) UBC, (**B**) HCK, and (**C**) UBA52.

Hydrogen bonding is a critical interaction force in protein–ligand binding, related to electrostatic interactions and reflecting their strength. The number of hydrogen bonds between Chicoric acid and protein UBC fluctuated primarily between 0 and 1 (Figure 15A), while with protein HCK, it fluctuated between 2 and 5 (Figure 15B). In contrast, the number of hydrogen bonds with protein UBA52 remained between 1 and 5 (Figure 15C).

##### Hydrogen Bond Occupancy (Frequency) Analysis

**Figure 16:**
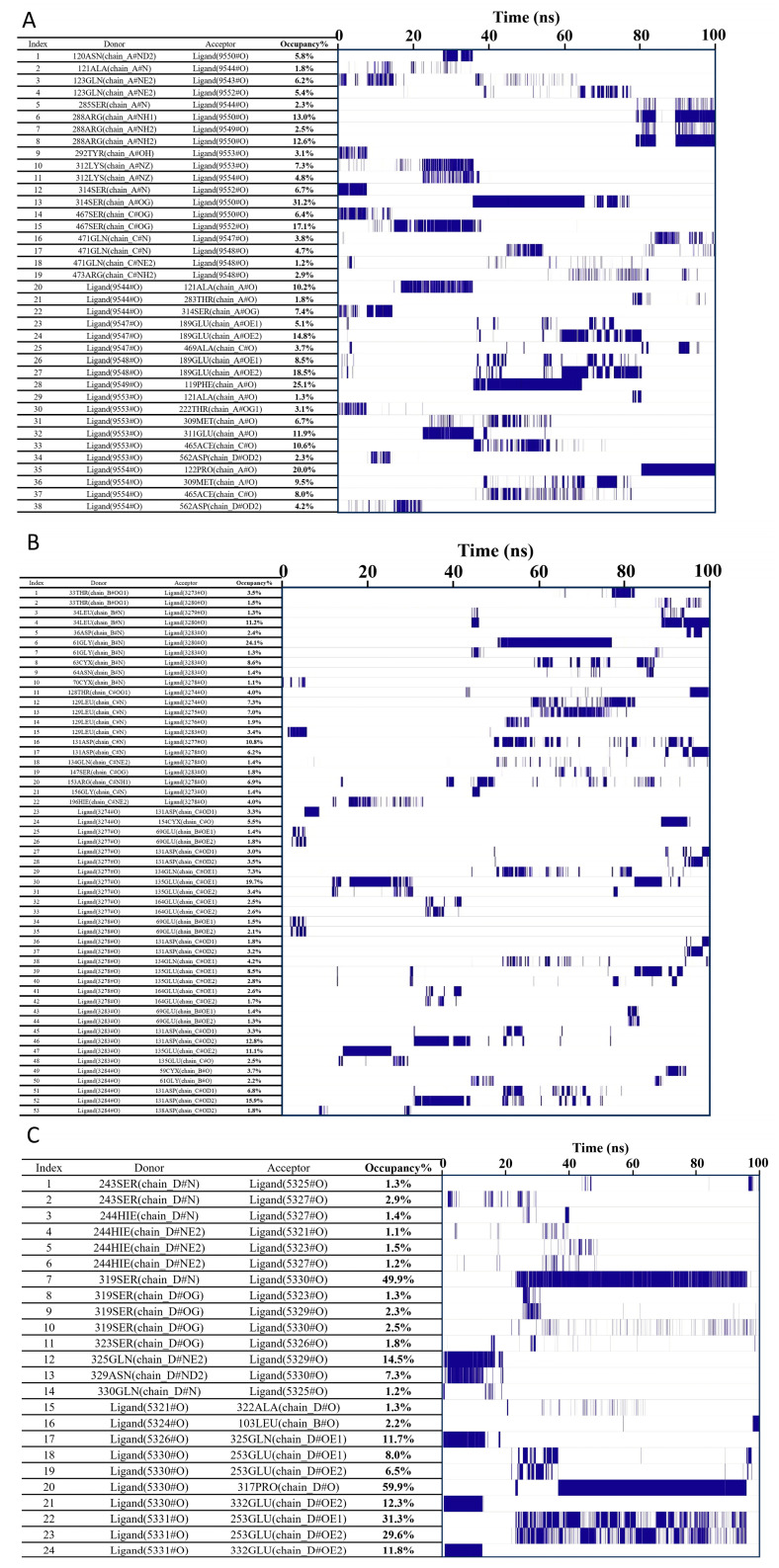
Hydrogen bond frequency analysis of molecular dynamics simulation results of UBC, HCK, and UBA52 with Chicoric acid: (**A**) UBC, (**B**) HCK, and (**C**) UBA52.

Hydrogen bond frequency analysis is a common method for studying the formation and persistence of intermolecular or intramolecular hydrogen bonds. It is used to determine the formation frequency, duration, and distribution of hydrogen bonds during the simulation. This analysis can identify high-frequency hydrogen bonds formed between the ligand and specific amino acid residues in the binding site, suggesting their critical role in the binding process. As shown in the figures, the left side displays the acceptor, donor, and occupancy of hydrogen bond pairs, while the right side shows the corresponding hydrogen bond formation frequency, with line density indicating the frequency level. According to Figure 16A–C, the hydrogen bonds formed between proteins UBC and HCK with Chicoric acid exhibit relatively low stability. In contrast, protein UBA52 and Chicoric acid demonstrate hydrogen bond pairs with higher stability, such as Ligand:317PRO, with an occurrence frequency of 59.9%, indicating strong stability.

#### 2.7.3. Analysis of Interactions Between the Small Molecule and Proteins

##### Analysis of Electrostatic and van der Waals Interactions

**Figure 17:**
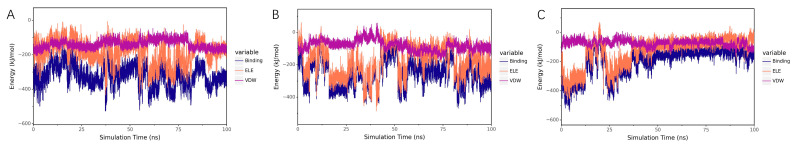
Electrostatic and van der Waals interaction analysis of molecular dynamics simulation results of UBC, HCK, and UBA52 with Chicoric acid: (**A**) UBC, (**B**) HCK, and (**C**) UBA52.

Without considering solvation effects, the van der Waals (VDW) and electrostatic (ELE) interactions between the small molecule and the protein in the complex were calculated to analyze changes in binding forces during the simulation. VDW represents van der Waals and hydrophobic interactions, while ELE denotes electrostatic interactions. The sum of VDW and ELE (binding) reflects the binding energy between the small molecule and the protein in the absence of solvation effects. As shown in Figure 17A–C, both VDW and ELE interactions in the three protein complexes (UBC, HCK, and UBA52) gradually stabilized during the simulation, indicating that the binding between the small molecule and the proteins became increasingly stable.

##### Binding Energy Analysis

Considering solvation energy and integrating RMSD, Rg, Distance, Buried SASA, and interaction energy, the stable-state trajectories of the complexes were selected. The MM-PBSA (Molecular Mechanics-Poisson Boltzmann Surface Area) method was employed to calculate the relevant energy terms of binding energy, as shown in Table 3a–c.

Among them, ΔEele represents the electrostatic interaction between the small molecule and the protein, ΔEvdw denotes the van der Waals interaction, ΔEpol indicates the polar solvation energy (reflecting electrostatic potential energy), and ΔEnonpol represents the non-polar solvation energy (reflecting hydrophobic interactions). ΔEMMPBSA is the sum of ΔEele, ΔEvdw, ΔEpol, and ΔEnonpol. The Gibbs binding energy ΔGbind is the sum of ΔEMMPBSA and −TΔS. (Note: Due to the significant computational error associated with -TΔS, this term is often excluded in binding energy comparisons, and ΔEMMPBSA is directly used as the binding energy. Additionally, the calculation of polar solvation energy ΔEpol also carries substantial uncertainty; thus, focus may be placed on other energy terms such as ΔEele, ΔEvdw, and ΔEnonpol.)

Based on the analysis of Table 3a, in the UBC protein complex, the van der Waals interaction energy (ΔEvdw) is higher than the electrostatic interaction energy (ΔEele), and both are higher than the hydrophobic interaction energy (ΔEnonpol). Therefore, in the composition of the binding energy, van der Waals interactions play a dominant role, electrostatic interactions play a secondary role, and hydrophobic interactions serve a supplementary role. The ΔEMMPBSA between the small molecule and the protein is −45.697 ± 1.277 kJ/mol, indicating high binding energy and strong affinity between them.

Based on the analysis of Table 3b, in the HCK protein complex, the van der Waals interaction energy (ΔEvdw) is higher than the electrostatic interaction energy (ΔEele), with van der Waals and electrostatic interactions being comparable in magnitude. Thus, in the composition of the binding energy, van der Waals interactions and electrostatic interactions play dominant roles, while hydrophobic interactions play a secondary role. The ΔEMMPBSA between the small molecule and the protein is −11.031 ± 5.742 kJ/mol, indicating relatively high binding energy and affinity between them.

Based on the analysis of Table 3c, in the UBA52 protein complex, the van der Waals interaction energy (ΔEvdw) is higher than the hydrophobic interaction energy (ΔEnonpol), while the electrostatic interaction energy (ΔEele) is comparable to the hydrophobic interaction energy. Therefore, in the composition of the binding energy, van der Waals interactions play a dominant role, while hydrophobic interactions and electrostatic interactions play secondary roles. The ΔEMMPBSA between the small molecule and the protein is −139.443 ± 2.563 kJ/mol, indicating high binding energy and strong affinity between them.

##### Residue Contribution Analysis

**Figure 18:**
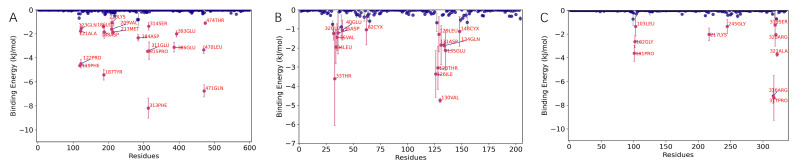
Residue contribution analysis of molecular dynamics simulation results of UBC, HCK, and UBA52 with Chicoric acid: (**A**) UBC, (**B**) HCK, and (**C**) UBA52.

The binding energy ΔEMMPBSA was decomposed to determine the contribution of each amino acid to the overall binding energy, thereby identifying critical residues in the protein. For the UBC protein, key amino acids contributing significantly to the binding energy include PHE-313 and GLN-471, as shown in Figure 18A. For the HCK protein, critical residues such as VAL-130 and THR-33 are highlighted in Figure 18B.

Similarly, for the UBA52 protein, key amino acids including PRO-317 and ARG-316 are illustrated in Figure 18C.

##### Structural Analysis

**Figure 19:**
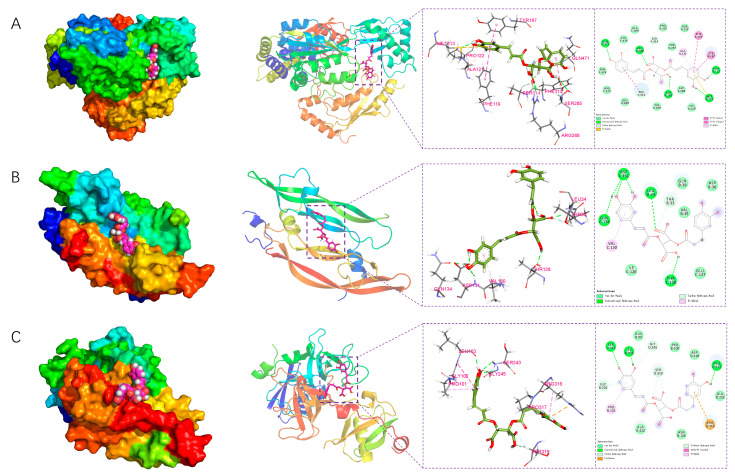
Structural analysis of molecular dynamics simulation results of UBC, HCK, and UBA52 with Chicoric acid: (**A**) UBC, (**B**) HCK, and (**C**) UBA52.

The conformation at the stable stage of the simulation was selected to analyze its structure and interactions. As shown in Figure 19A, amino acids PRO-122, MET-213, ARG-288, and GLN-471 in the UBC protein formed hydrogen bonds with the small molecule. MET-213, PHE-119, TYR-187, ALA-121, and PHE-313 engaged in hydrophobic interactions with the small molecule, including Pi–Sulfur, Pi-Pi Stacked, Pi-Pi T-shaped, and Pi–Alkyl interactions. Amino acids such as GLN-123 and ARG-473 participated in van der Waals interactions with the small molecule.

As illustrated in Figure 19B, the small molecule interacts with the HCK protein through multiple forces. It forms conventional hydrogen bonds with ASP-131, LEU-34, GLN-134, and THR-128. Additionally, a Pi-alkyl interaction is observed with VAL-130, while other residues, including ASP-36 and ILE-126, participate through van der Waals contacts.

As shown in Figure 19C, amino acids PRO-317, LEU-103, and SER-243 in the UBA52 protein formed hydrogen bonds with the small molecule. ARG-316 and PRO-101 engaged in hydrophobic interactions with the small molecule, including Pi-Cation, Amide-Pi Stacked, and Pi-Alkyl interactions. Amino acids such as GLN-99 and ASN-104 participated in van der Waals interactions with the small molecule.

## 3. Discussion

Currently, the application of network pharmacology technology has achieved promising results in predicting drug targets and studying their mechanisms. Huang Can et al. [20], in their study on the mechanism of action of cepharanthine against SARS-CoV-2, utilized PubChem, the DAVID database, the STRING database, and the molecular docking method to predict key proteins. They identified 40 intersection targets between cepharanthine and COVID-19, with key targets primarily involving Src, AKT1, EGFR, and mTOR, among others. Jin Yaqing et al. [21], in their study on the mechanism of Elsholtzia splendens in treating influenza, utilized databases such as TCMSP, GeneCards, OMIM, DAVID, and STRING to identify nine core proteins including Akt serine/threonine kinase 1 (Akt1), non-receptor tyrosine kinase (SRC), heat shock protein (HSP) 90AA1, epidermal growth factor receptor (EGFR), matrix metalloproteinase 9 (MMP9), prostaglandin–endoperoxide synthase 2 (PTGS2), and mechanistic target of rapamycin (mTOR). These findings suggest that Elsholtzia splendens may exert anti-influenza effects through multiple mechanisms, including direct antiviral activity, immunomodulation, and anti-inflammatory actions. Li Zhi et al. [22], in their study on the mechanism of baicalein against porcine deltacoronavirus (PDCoV) infection, identified common targets of baicalein and PDCoV infection through databases such as PharmMapper, PubChem, STRING, TCMSP, and Swiss Target Prediction. Their findings indicated that baicalein’s anti-PDCoV effects exhibit multi-target and multi-pathway characteristics, potentially acting on core genes including AKT1, HSP90AA1, SRC, EGFR, CASP3, MAPK, and STAT3 to regulate the PI3K-Akt, Ras, and MAPK signaling pathways, apoptosis, viral infection, and other processes.

This study employed the PharmMapper, GeneCards, and OMIM databases to identify 31 common candidate targets shared between Chicoric acid and the influenza virus. Based on 31 candidate targets, a protein–protein interaction (PPI) network was constructed using the STRING and Cytoscape databases, and five core targets—UBA52, UBC, HCK, CDKN1B, and RPS27A—were screened based on their degree, betweenness centrality, and closeness centrality.

Both UBA52 and UBC function as essential precursors for intracellular free ubiquitin and play critical roles in maintaining the integrity and functionality of the ubiquitin–proteasome system. Deficiency in UBA52 has been associated with cell cycle arrest, impaired DNA repair capacity, and diminished protein synthesis [23,24]. Notably, Qiao W et al. [25] reported that the host protein UBA52 interacts with H5N1 avian influenza viral proteins PA, PAN155, and PAN182, thereby modulating viral replication. UBC is recognized as a canonical heat shock response protein, whose transcriptional upregulation under proteotoxic stress—including heat shock or proteasome inhibition—is primarily mediated by the binding of heat shock factor 1 (HSF1) to the heat shock element (HSE) located in its promoter region [26,27]. Supporting this mechanism, Bianchi M et al. [28] demonstrated that Nrf2 knockdown in HeLa cells does not alter stress-induced UBC expression, whereas arsenite-induced stress enhances UBC expression in an HSF1-dependent but Nrf2-independent manner. This study proposes that Chicoric acid, owing to its structural resemblance to ubiquitin or ubiquitin-binding domains (e.g., UBA, UBD), may competitively interact with UBA52 and UBC proteins. Such an interaction could occupy the molecular interfaces required for their association with E3 ubiquitin ligases or substrate proteins, potentially disrupting the stability and functional dynamics of key proteins involved in the influenza virus life cycle. Concurrently, this interference may exacerbate cell cycle arrest resulting from UBA52 dysfunction, thereby indirectly suppressing viral replication, which depends on active host cell cycling.

HCK, a member of the Src family of kinases, is predominantly expressed in myeloid cells and plays a pivotal role in regulating inflammatory responses and cell migration. It modulates key cellular processes through activation of the NLRP3 inflammasome [29], induction of macrophage M2 polarization [30], and regulation of EGFR expression [31]. This study proposes that Chicoric acid may act as a potent inhibitor of HCK kinase activity, potentially through competitive binding to the ATP-binding pocket or interaction with allosteric regulatory sites, thereby disrupting downstream signaling pathways involved in viral propagation and inflammation. Given that HCK substrates are critically involved in cytoskeletal reorganization—mediating viral endocytosis—and in inflammatory signal transduction, including NF-κB pathway activation, HCK inhibition is likely to confer dual antiviral benefits: suppression of viral entry during the early stages of infection and attenuation of excessive host inflammatory responses in later phases. This mechanistic rationale is consistent with the broad-spectrum antiviral activity demonstrated by established Src family kinase inhibitors such as Dasatinib [32,33], offering a plausible explanation for the observed pharmacological effects of Chicoric acid and further supporting the therapeutic potential of targeting Src family kinases in antiviral interventions.

CDKN1B is a key cyclin-dependent kinase inhibitor that negatively regulates cell proliferation by arresting the cell cycle at the G1 phase and participates in the regulation of apoptosis [34,35,36,37,38,39]. Decreased expression of CDKN1B has been linked to poor prognosis in multiple types of tumors [40,41]. We hypothesize that Chicoric acid may directly bind to the CDKN1B protein, stabilizing its conformation and protecting it from degradation via phosphorylation- and ubiquitination-dependent pathways triggered by viral or oncogenic proteins. The resulting accumulation of CDKN1B protein is expected to induce G1 phase cell cycle arrest, thereby preventing viruses from entering the S phase and exploiting replication resources available during this stage. This process ultimately leads to the suppression of viral genome replication and proliferation.

RPS27A is a non-conventional ribosomal protein that serves not only as a component of the ribosome but also as a ubiquitin precursor (UBA80), independently participating in cell cycle regulation, apoptosis, and transcriptional control [42,43,44]. It can induce cell cycle arrest via the Raf/MEK/ERK pathway and stabilize p53 by suppressing MDM2 [45,46]. RPS27A is highly expressed in various cancers and virus-infected liver tissues [47,48,49,50,51]. We propose that Chicoric acid disrupts RPS27A function through two synergistic mechanisms: First, it directly targets RPS27A, impairing its role in ribosome biogenesis or translation initiation, thereby globally reducing host protein synthesis and directly inhibiting rapid viral protein production. Second, Chicoric acid interferes with the maturation of RPS27A into ubiquitin, disturbing the intracellular free ubiquitin equilibrium. This latter effect may act in concert with the inhibitory action of Chicoric acid on UBA52/UBC, amplifying the overall disruption of the ubiquitin–proteasome system. Together, these actions suppress viral proliferation by simultaneously targeting viral protein synthesis and host cell homeostasis.

To further validate the network pharmacology findings, molecular docking and molecular dynamics simulations were employed to evaluate the binding affinity and stability of the complexes between the screened protein targets and Chicoric acid at the molecular level, under both static and dynamic conditions. According to Sun et al. [52], a binding energy below 0 kJ/mol suggests spontaneous binding, while a value lower than −20 kJ/mol indicates stable complex formation. Our molecular docking results demonstrated that all five core proteins bound spontaneously to Chicoric acid, with binding energies below 0 kJ/mol. Among them, UBC and UBA52 exhibited the lowest values.

Molecular dynamics (MD) simulations were performed on the top three protein targets—UBC, HCK, and UBA52—selected based on molecular docking binding energies. The simulation results further validated the feasibility of the docking-based conclusions. In this study, 100 ns MD simulations were conducted to systematically evaluate the binding characteristics and conformational dynamics of Chicoric acid with UBA52, HCK, and UBC. Analysis of the MD trajectories indicated that all three protein–Chicoric acid complexes maintained good structural stability throughout the simulation. Root mean square deviation (RMSD) analysis revealed that the UBC complex reached equilibrium rapidly within 10 ns and remained stable at approximately 0.25 nm. In contrast, UBA52 and HCK underwent brief conformational adjustments before stabilizing at around 0.2–0.4 nm and 0.3 nm, respectively. Notably, the RMSD profiles of Chicoric acid closely matched those of the corresponding protein backbones in all systems, suggesting a stable binding mode without significant displacement or flipping. Local flexibility, assessed by root mean square fluctuation (RMSF), indicated elevated fluctuations in non-binding regions (e.g., residues 50–70, 150–170, and 250–280 in UBA52; 40–60, 120–140, and 170–190 in HCK; 150–170 and 380–400 in UBC). However, residues within the binding pockets exhibited markedly reduced RMSF values, indicating that Chicoric acid binding restricted local structural fluctuations and exerted a stabilizing effect at the binding site. Structural compactness and hydrophobic core stability further supported these observations [53]. The radius of gyration (Rg) values for UBA52, HCK, and UBC stabilized within narrow ranges of 2.15–2.25 nm, 2.04–2.08 nm, and 2.55–2.61 nm, respectively, indicating that ligand binding did not compromise overall structural compactness. Moreover, minimal variation in the solvent-accessible surface area (SASA) suggested tight packing of hydrophobic cores without significant exposure due to structural relaxation. Binding free energy calculations indicated that Chicoric acid affinity followed the order UBA52 > UBC > HCK, with electrostatic interactions (ELE) serving as the primary driving force. The contribution of ELE increased with higher binding affinity. Hydrogen bond analysis and binding distance measurements further highlighted differences in binding stability: UBA52 and UBC complexes maintained 0–1 and 1–5 hydrogen bonds, respectively, with binding distances under 0.3 nm, whereas the HCK system formed 2–5 hydrogen bonds with distances slightly exceeding 0.3 nm. Energy decomposition per residue revealed distinct binding mechanisms across the proteins: in HCK, Chicoric acid interacted mainly with acidic residues such as GLU40 and ASP36; in UBC, polar residues ASP28 and GLU311 dominated; and in UBA52, basic residues ARG316, ARG320, and LYS217 contributed most significantly—suggesting potential salt bridge formation with the carboxyl groups of Chicoric acid, consistent with the highest binding affinity in this system. Hydrophobic residues, including LEU29 (HCK), PHE313 (UBC), and LEU103 (UBA52), also participated in ligand binding across all systems, indicating a general role for hydrophobic effects in stabilizing the binding mode. Analysis reveals that Chicoric acid exhibits a conserved binding mechanism across UBA52, HCK, and UBC, defined by a synergistic pattern of dominant electrostatic interactions, complemented by hydrogen bonding and hydrophobic contributions. Notwithstanding this shared mechanism, pronounced differences are observed in the specific residue involvement and binding conformations.

## 4. Materials and Methods

### 4.1. Screening of Chicoric Acid Targets

The 3D structure of Chicoric acid (CAS: 70831-56-0) was obtained from the PubChem database (https://pubchem.ncbi.nlm.nih.gov (accessed on 19 April 2024)) [54] in SDF format and submitted to the PharmMapper (http://www.lilab-ecust.cn/pharmmapper/ (accessed on 19 April 2024)) [55] server for analysis. The submission employed the “Druggable Pharmacophore Models” set, with the parameter for the number of matched targets set to 300, which returned the top 300 potential targets based on fit score. A subsequent query of these target names using the UniProtKB search function in the UniProt database converted all identifications to their official gene symbols (Official Symbols) for consistency.

### 4.2. Screening of Influenza Targets

Retrieval was performed using the keyword “influenza virus” in the GeneCards database (https://www.genecards.org/ (accessed on 21 April 2024)) [56] and the OMIM database (https://omim.org/ (accessed on 1 May 2024)) [57,58] to acquire and screen target data related to influenza. The targets screened from both disease databases were processed by removing duplicate entries, resulting in a set of targets associated with influenza virus.

### 4.3. Visual Analysis of Chicoric Acid Against Influenza Virus

A Venn diagram was plotted using the online bioinformatics platform (http://www.bioinformatics.com.cn/ (accessed on 10 May 2024)) [59] to visualize the anti-influenza virus effects of Chicoric acid.

### 4.4. Construction and Analysis of the Protein–Protein Interaction Network

The protein–protein interaction (PPI) network for the potential targets of Chicoric acid against influenza was constructed using the STRING database (https://string-db.org/ (accessed on 11 May 2024)) [60], with the organism limited to Homo sapiens and an interaction score threshold set to medium confidence (0.400). The resulting TSV file from STRING was imported into Cytoscape 3.9.1 [61] for network visualization and analysis. Subsequently, the CentiScaPe 2.2 application analyzed this network to screen for the core targets based on topological algorithms.

### 4.5. GO Functional Enrichment Analysis and KEGG Pathway Analysis

Access the DAVID database (https://david.ncifcrf.gov/) [62], import the potential targets of Chicoric acid against influenza, and select the species “Homo sapiens”. Set the “Select Identifier” to “OFFICIAL_GENE_SYMBOL” and the “List Type” to “Gene List”. Perform GO analysis and KEGG [63] pathway analysis on the targets of Chicoric acid against influenza, and export the results as a TXT file. Screening was conducted according to *p* < 0.05. Arranged in ascending order, bar charts were drawn for the top 10 biological processes in terms of the number of targets in the three branches of GO, namely biological processes, molecular functions, and cellular components. Use the R package in Bioconductor to draw bubble charts for the top 20 KEGG pathways in terms of the number of targets.

### 4.6. Construction of the Drug–Target–Pathway Network

Access Cytoscape 3.9.1 software, import the targets and pathway information related to Chicoric acid’s anti-influenza activity, and construct a “drug–target–pathway” network diagram.

### 4.7. Molecular Docking Validation of Chicoric Acid with Targets

Access the PubChem database to download the structure of the ligand Chicoric acid and save it in SDF format. Use Open Babel to convert it to MOL2 format. Open the small ligand molecule with AutoDock Tools 1.5.6 [64,65]; then, add hydrogen atoms, assign charges, detect the root of the ligand, perform rotatable bond search and definition, and save the file in PDBQT format. Access the RCSB Protein Data Bank to download the 3D structure of the core target protein. In AutoDock Tools 1.5.6, perform molecular docking between Chicoric acid and the important target proteins. After steps such as dehydration and adding hydrogen, use the Auto Grid and Auto Dock modules to perform the docking and obtain the binding affinity. Use Open Babel 2.4.1 software to convert the molecular docking results from the aforementioned PDBQT format files into PDB files. Select the conformation with the lowest docking binding energy for binding mode analysis, and use PyMOL to visualize the molecular docking results, plot intermolecular hydrogen bonds, and add labels.

### 4.8. Molecular Dynamics Simulation

To further investigate the stability of the binding between the small ligand molecule and the large protein molecule, molecular dynamics simulations were performed on the protein receptors with high binding energy to the ligand molecule using the software. The molecular dynamics (MD) simulations were conducted using the Gromacs 2022 [66,67] program. The GAFF force field was applied to the small molecule, while the AMBER14SB force field and TIP3P water model were used for the protein. The files of the protein and the small-molecule ligand were merged to construct the simulation system for the complex. The simulations were performed under constant temperature and pressure with periodic boundary conditions. During the MD simulation, all involved hydrogen bonds were constrained using the LINCS algorithm, with an integration time step of 2 fs. Electrostatic interactions were calculated using the Particle-mesh Ewald (PME) method, with a cutoff set to 1.2 nm. The non-bonded interaction cutoff was set to 10 Å and updated every 10 steps. The V-rescale temperature coupling method was employed to maintain the simulation temperature at 298 K, while the Berendsen method was used to control the pressure at 1 bar. At 298 K, 100 ps of NVT and NPT equilibrium simulations were performed, followed by a 100 ns MD simulation of the complex system, with conformations saved every 10 ps. After the simulation, VMD and PyMOL were used to analyze the simulation trajectory, and the g_mmpbsa program was applied to calculate the MMPBSA binding free energy between the protein and the small-molecule ligand.

## 5. Conclusions

In summary, this study systematically elucidated the molecular mechanisms through which Chicoric acid exerts anti-influenza effects via multi-target and multi-pathway synergy, employing an integrated computational strategy that combined network pharmacology, molecular docking, and molecular dynamics simulations. The results demonstrate that Chicoric acid exhibits stable binding affinity with key targets such as UBC and UBA52. The structural basis of these interactions was characterized at the atomic level, providing a theoretical foundation for developing Chicoric acid as a novel multi-target anti-influenza agent.

However, it should be noted that although the integrated computational approach adopted in this study enabled a shift from a single-target to a network-level perspective, enhanced screening efficiency from broad to focused analysis, and offered deeper mechanistic insights by transitioning from static binding evaluation to dynamic simulation, certain limitations remain. For instance, network pharmacology analyses are constrained by the completeness and accuracy of underlying databases, which may introduce systematic bias. Computational simulations, as simplified models of complex biological systems, cannot fully recapitulate actual binding conformations or dynamic processes in vivo. Most importantly, in the absence of experimental validation, the findings of this study remain hypothetical, and their biological relevance and translational potential require further confirmation.

To address these limitations, future research should focus on integrating computational predictions with rigorously designed experimental studies. Key targets and pathways should be systematically verified using in vitro antiviral assays, cellular molecular biology techniques, and animal models. Further efforts should aim to clarify the specific sites of action of Chicoric acid within multiple pathways and to quantify the relative contributions of individual targets to the observed synergistic effects. Moreover, incorporating multi-omics data to develop predictive models that more accurately reflect real biological systems will improve the reliability of computational strategies and facilitate the translation of Chicoric acid into preclinical research.

## Figures and Tables

**Table 1:** Anti-influenza virus targets of Chicoric acid.

Serial Number	Uniport ID	Gene	Name
1	Q9Y6I3	*EPN1*	Epsin-1
2	Q29122	*MYO6*	Unconventional myosin-VI
3	P52948	*NUP98*	Nuclear pore complex protein Nup98-Nup96
4	P12004	*PCNA*	Proliferating Cell Nuclear Antigen
5	P63000	*RAC1*	Rac Family Small GTPase 1
6	P02788	*LTF*	Lactotransferrin
7	P55735	*SEC13*	Protein SEC13 homolog
8	P35997	*RPS27A*	Small ribosomal subunit protein eS27A
9	P62987	*UBA52*	Ubiquitin–ribosomal protein eL40 fusion protein
10	P0CG47	*UBB*	Polyubiquitin-B
11	P0CG48	*UBC*	Polyubiquitin-C
12	P12318	*FCGR2A*	Fc Gamma Receptor IIa
13	P05979	*PTGS1*	Prostaglandin G/H synthase 1
14	Q9UKF6	*CPSF3*	Cleavage And Polyadenylation Specific Factor 3
15	P46527	*CDKN1B*	Cyclin-Dependent Kinase Inhibitor 1B
16	P78536	*ADAM17*	ADAM Metallopeptidase Domain 17
17	P06681	*C2*	Complement C2
18	P19878	*NCF2*	Neutrophil cytosol factor 2
19	P00156	*MT-CYB*	Cytochrome b
20	P00491	*PNP*	Purine nucleoside phosphorylase
21	P01008	*SERPINC1*	Antithrombin-III
22	P08631	*HCK*	Tyrosine–protein kinase HCK
23	P08069	*IGF1R*	Insulin-like growth factor 1 receptor
24	Q02548	*PAX5*	Paired box protein Pax-5
25	Q8WYN0	*ATG4A*	Cysteine protease ATG4A
26	P07288	*KLK3*	Prostate-specific antigen
27	Q99259	*GAD1*	Glutamate decarboxylase 1
28	Q8NI35	*PATJ*	InaD-like protein
29	Q5T0N5	*FBP1*	Formin-binding protein 1-like
30	P05198	*EIF2S1*	Eukaryotic Translation Initiation Factor 2 Subunit Alpha
31	P11181	*DBT*	Lipoamide acyltransferase component of branched-chain alpha-keto acid dehydrogenase complex, mitochondrial

**Table 2:** Results of docking with Chicoric acid.

Gene	PDB ID	Free Energy of Binding/(kJ/mol)
*UBC*	6ULH	−21.57
*HCK*	6Z3F	−20.69
*UBA52*	7OWC	−19.31
*RPS27A*	6SQR	−13.69
*CDKN1B*	6P8G	−12.46

**Table 3:** (**a**). Binding energy and its components for the UBC protein in a stable state. (**b**). Binding energy and its compositional breakdown for the HCK protein under stable conditions. (**c**). Binding energy and its components for the UBA52 protein under stable-state conditions.

Complex	ΔEvdw	ΔEele	ΔEpol	ΔEnonpol	ΔEMMPBSA	−TΔS	ΔGbind *
(**a**)							
Protein–Ligand	−181.42 ± 2.57	−72.79 ± 8.44	233.84 ± 10.26	−25.33 ± 0.13	−45.70 ± 1.28	27.76 ± 1.34	−17.94 ± 2.57
(**b**)							
Protein–Ligand	−105.75 ± 3.19	−99.40 ± 5.31	213.29 ± 4.44	−19.17 ± 0.25	−11.03 ± 5.74	33.40 ± 3.58	22.37 ± 4.23
(**c**)							
Protein–Ligand	−159.84 ± 2.73	−18.89 ± 7.83	60.75 ± 8.12	−21.47 ± 0.09	−139.44 ± 2.56	19.92 ± 2.25	−119.52 ± 1.67

Note: * ΔGbind = ΔEvdw + ΔEele + ΔEpol + ΔEnonpol − TΔS.

## Data Availability

Data are contained within the article.
